# A pilot investigation of the efficacy and safety of magnesium chloride and ethanol as anesthetics in *Loligo vulgaris* embryos

**DOI:** 10.3389/fphys.2022.968047

**Published:** 2022-09-14

**Authors:** Marta Sprecher, Simon G. Sprecher, Claudia Spadavecchia

**Affiliations:** ^1^ Department of Biology, University of Fribourg, Fribourg, Switzerland; ^2^ Department of Clinical Veterinary Medicine, Anaesthesiology and Pain Therapy Section, Vetsuisse Faculty, University of Bern, Bern, Switzerland

**Keywords:** anesthesia, cephalopods embryos, *Loligo vulgaris*, ethanol, administration and dosage, magnesium chloride (MgCl_2_)

## Abstract

The inclusion of cephalopods in the legislation related to the use of animals for experimental purposes has been based on the precautionary principle that these animals have the capacity to experience pain, suffering, distress, and lasting harm. Recent studies have expanded this view and supported it. Handling cephalopod mollusks in research is challenging and whenever more invasive procedures are required, sedation and/or anesthesia becomes necessary. Therefore, finding adequate, safe, and effective anesthetics appears mandatory. Several substances have been considered in sedating cephalopods, in some instances applying those utilized for fish. However, species-specific variability requires more detailed studies. Despite long-lasting experience being linked to classic studies on squid giant axons, evidence of action on putative anesthetic substances is scarce for *Loligo vulgaris* and particularly for their embryos. The aim of the current study was to evaluate effects elicited by immersion of squid embryos in anesthetic solutions and examine whether these forms display a similar reaction to anesthetics as adults do. Different concentrations of ethanol (EtOH; 2, 2.5, and 3%) and magnesium chloride (MgCl_2_; 1, 1.5, and 1.8%) were tested by adopting a set of indicators aimed at exploring the physiological responses of squid embryos. Forty-two embryos of the common squid *Loligo vulgaris* (stages 27–28) were assigned to three conditions (EtOH, MgCl_2,_ and controls) and video recorded for 15 min (5 min before, 5 min during, and 5 min after immersion in the anesthetic solutions). In each group, the heart rate, respiratory rate, buoyancy, chromatophore activity, and tentacles/arms responses were assessed to evaluate the embryos' vitality and responsiveness to stimulation. Both substances provoked a decrease in heart and respiratory rates and inhibited buoyancy, chromatophores, and tentacles/arms responses; no adverse effects were observed. EtOH had a faster onset of action and faster recovery than MgCl_2_, being potentially more adequate as an anesthetic for shorter procedures. Even though MgCl_2_ caused a longer muscle relaxation, the reversibility was not confirmed for the 1.8% concentration; however, lower concentrations triggered similar results as the ones obtained with the highest EtOH concentrations. We have shown that the late developmental stages of *Loligo vulgaris* embryos could represent a good model to evaluate anesthetics for cephalopods since they can display similar reactions to anesthetics as adults animals do.

## Introduction

In the last decade, cephalopod mollusks have been included in European Union regulation for the use of live animals by transposition into the national legislation of the Directive 2010/63/EU “on the protection of animals used for scientific purposes.” In Switzerland, the Animal Protection Ordinance (Schweizerische-Tierschutzverordnung, 2018) includes cephalopods in Chapter 1 (General Provisions—Aims) and Chapter 6 (Experiments on animals) to be protected by law since 2018. In particular, “Chapter 1” defines the categories of animals that will be protected by the ordinance; “Chapter 6” deals with animal experimentation and defines three types of cephalopods according to their sizes (small, medium, and large), no species-specific recommendation has been provided. The inclusion was based on the precautionary principle (Birch, 2017, 2021) that these animals possess the capacity to experience pain, suffering, distress, and lasting harm, further supported by some more recent findings (EFSA Panel, 2005; Crook et al., 2011; Crook and Walters, 2011; Andrews et al., 2013; Crook et al., 2013; Crook, 2021). Over the last years, an increasing number of studies have been focused on various aspects of the biology, physiology, and ecology of cephalopods. This induced an expansion of the number of species studied and also an increase in the number of studies. Therefore, an increase in attention toward the welfare of these animals is mostly required by the scientific community as well as fisheries. It is remarkable to mention that in the EU Member States, the number of cephalopods used in research in 2018 accounted for over four thousand animals according to (EU, 2019).

To perform animal handling such as for hemolymph withdrawal (Jozet-Alves et al., 2008; Locatello et al., 2013), surgeries such as limb amputation for biomechanical studies, pain or nerve regeneration studies (Imperadore et al., 2019b; Howard et al., 2019; Nesher et al., 2019), or tissue sampling (Mackie, 2008; Ponte and Fiorito, 2015; Zatylny-Gaudin et al., 2016; Baldascino et al., 2017; Imperadore et al., 2019a), the animal should be anesthetized. In mammals, the application of sedation protocols must ensure the “AALLR,” meaning analgesia, amnesia, loss of consciousness, loss of control reflexes, and muscle relaxation (Katzung et al., 2021). In cephalopods and, more particularly, in the common squid *Loligo vulgaris*, other criteria are needed to define satisfactory anesthesia, since their physiological characteristics are unique and cannot be easily compared to those of mammals under similar circumstances (Sen and Tanrikul, 2009). Previous work of various laboratories have urged the use of standardized protocols with specific criteria to evaluate satisfactory anesthesia in cephalopods: ventilation rate, chromatophore coloration change, loss of sucker adhesiveness, loss of voluntary or provoked movement, and unresponsiveness to noxious stimuli (Pagano et al., 2011; Gleadall, 2013; Butler-Struben et al., 2018). On the other hand, the major criteria to be observed during sedation on the basis of those used in other aquatic animals (McFarland and Klontz, 1969) include breathing depression, pupil constriction due to flash of light, reaction to pinching eye skin, flabbiness of arms, and the lack of reaction to stimulus. But are the same parameters used for adult animals also comparable to those used for embryos? Do cephalopod embryos display similar reactions as their adults do during anesthesia?

To date, ethanol (EtOH) and magnesium chloride (MgCl_2_) have been the most used substances to anesthetize cephalopods (Messenger et al., 1985; Butler-Struben et al., 2018; Abbo et al., 2021). Indeed, according to a recent review, out of 48 studies in which procedures for “anesthetizing” cephalopods were mentioned, 17 reported MgCl_2_, 23 EtOH in seawater, 3 a combination of both, and only 5 a different agent (Fiorito et al., 2015). The wide acceptance of EtOH and MgCl_2_ is linked to the fact that these substances are easy to use, not reported to be toxic for the user, and do not require special permits for purchase (depending on the country). Furthermore, they are cheap and considered more “eco-friendly” than other substances that have been suggested to be utilized with cephalopods (e.g., isoflurane; Polese et al., 2014) where special equipment is needed, and particularly, attention to disposal is required (Kumar et al., 2013; Varughese and Ahmed, 2021).

Detailed information about the physiological effects of EtOH and MgCl_2_ in cephalopods remains scarce, especially for *Loligo vulgaris* and, more particularly, for embryos at their latest stages. Most of the recent studies on *Loligo* spp. embryos are either of ecological interest or related to development (Burbach et al., 2014; Rosa et al., 2014; Crawford et al., 2020). Therefore, it is important to not only establish anesthesia protocols but also assess whether the same indicators used for adults may be applied to embryos and determine if embryos respond in the same way to the anesthetics as adults do.

Late-stage embryos provide an interesting life stage to explore, considering that their nervous system is well developed and their capacity to learn also appears weeks before hatching as shown for *Sepia* embryos (Darmaillacq et al., 2006; Lee et al., 2020). Another important fact is that embryos can be available in reasonable numbers.

Here, we utilized *L. vulgaris* embryos to evaluate responses to immersion in EtOH and MgCl_2_ solutions and assess the adequacy of these substances as anesthetics. In our aims, we 1) provide baseline heart rate and respiratory rate data for embryos; 2) describe changes in the heart and respiratory rates, buoyancy, chromatophore activity, and tentacle/arms responses induced by immersion in EtOH (2, 2.5, and 3% in seawater) and MgCl_2_ (1, 1.5, 1.8% in seawater); 3) compare the responses to anesthetic solutions of squid embryos with the adult data from other studies; and 4) assess the reversibility of anesthesia on the embryos. To the best of our knowledge, our study provides a first baseline and reference for physiological parameters of respiration and heart rate of late-stage *L. vulgaris* embryos.

## Materials and methods

### Sample collection, housing, and husbandry

Eggs from the species *Loligo vulgaris* were collected during a sampling project of the European Marine Biological Resource Center (EMBRC) France at the Bay of Morlaix on the 22 January 2021. The eggs which are normally attached to a substrate were found in a rocky sandy habitat. The eggs were transported by a commercial courier in 30-L plastic bags with one-thirds seawater and two-thirds oxygen following the procedure by Fuentes et al. (2005). The duration of the transport was 24 h at a temperature of 14°C, and the density was three branches per bag (i.e., less than 3,000 embryos/bag).

**FIGURE 1:**
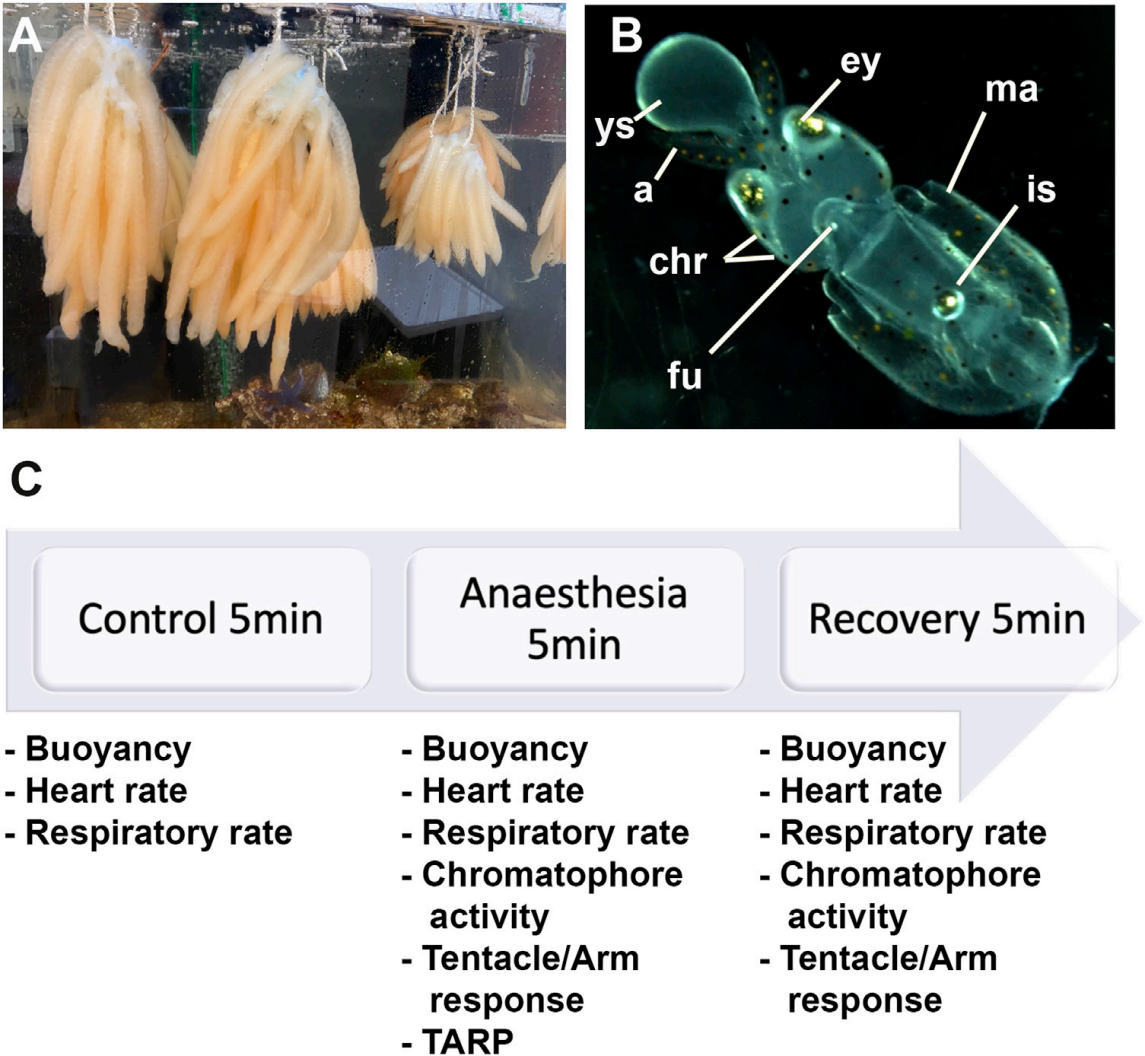
**(A)** Eggs of *Loligo vulgaris* suspended in bundles; each bundle contains between 15 and 30 arms. Each arm contains between 100 and 150 embryos. **(B)** Embryo stages 27–28; ys: external yolk sac, a: arms, ey: eyes, chr: chromatophores, fu: funnel, ma: mantel, is: ink sac. **(C)** Experiment timeline.

Upon arrival in the laboratory, the eggs were incubated in a closed artificial seawater system [Red Sea REEFER 170 Deluxe Aquarium (Black)]. The tank contained 130 L of artificial seawater at a temperature of 14 ± 0.5°C (salinity range: 34–36 ppt; pH range: 7.99–8.13). A 12-h light/dark cycle was set with a crepuscular and a dawn light regime, and Maxspect éclairage LED (RSX 100W) was utilized. The eggs were suspended in bundles (see Figure 1A) in a plastic structure made with PVC. Each bundle comprised between 15 and 30 arms. Each arm hosted between 100 and 150 embryos. Taking into account that the oxygen consumption of one egg mass of octopus is twice the consumption of an adult of an average weight size (Parra et al., 2000) and to provide the best care to warrant adequate care, squid eggs' oxygenation was further facilitated by adding two additional bubblers with air diffusers (Tetra APS 100 and Tetratec AS45).

### Experimental setup and embryos

Each procedure was conducted by the same person and under the same conditions. The observations of the embryos were carried out using a stereoscope (Leica Microsystems; Leica Plan APO 1.0×, 10450028; CLS100 LED).

We utilized *L. vulgaris* embryos at developmental stages 27–28 that were identified following the procedure by Arnold et al. (1974) (Figure 1B). At this stage, the yolk matches the length of the arms or the head; the latter representing one-third of the mantle length. Hatching could occur if stimulated artificially. At this stage, the ink sac is pigmented.

The eggs were placed in a Petri dish containing artificial seawater. Salinity and temperature were kept constant at 34 ppt and 14°C, respectively. The eggs were dissected carefully with a forceps (F. S. T, no. 11254-20), and the hatchlings were placed in a three-compartment culture glassware containing artificial seawater (salinity: 34 ppt; temperature: 14°C; formulation of the artificial seawater was 4 kg of artificial sea salt per 100 L of deionized water). The individuals were chosen randomly from three different egg branches; no sex differentiation was performed. The egg cases were dissected to remove the embryos, and once the embryos were out of their cases, no further development was observed.

Samples were randomly attributed to three groups. The first group (N = 18 embryos; EtOH group) served to test three different EtOH concentrations; the second group (N = 18, MgCl_2_ group) was used to test three different concentrations of MgCl_2_; and the third group represented the control. Each embryo was tested only for one concentration. Anesthetic solutions were made in artificial seawater, and we did not determine any potential difference in the osmotic pressure of the solutions utilized in the different treatments.

### Anesthesia protocol

We identified three phases (5 min each): the control phase (t0), the anesthesia phase (t1), and the recovery phase (t2; Figure 1C). According to the Guidelines for the Care and Welfare of Cephalopods in Research, different times were proposed for anesthesia purposes for different species, but no references exist for *Loligo* and specifically for embryos (Fiorito et al., 2015). The averages suggested were between 8 and 19 min while using a single substance and according to the body size. In addition, the findings of Marking and Meyer (1985) regarding aquatic animals propose 3 min for sedation and 5 min for recovery. Therefore, we decided to use 5 min for each procedure. During the first 5 minutes (control phase; t0), the health status of the embryos was assessed. Then, artificial seawater was removed by a pipette and immediately replaced with the anesthetic solutions (anesthesia phase; t1). Afterward, the anesthetic was changed back to fresh artificial seawater (recovery phase, t2). During the three phases, the heart and respiratory rates were recorded, while the remaining parameters were evaluated only during t1 and t2. We recorded

- heart rate “hr”;
- respiratory rate “rr”;
- time until loss of buoyancy “TL B” and time to recover buoyancy “TR B″;
- time until loss of chromatophores activity “TL CR” and time to recover chromatophores activity “TR CR”;
- time until loss of tentacles/arms response “TL T/A R” and time to recover tentacles/arms response “TR T/A R″; and
- typical adverse response pattern “TARP” (as defined by Gleadall, 2013).

Anesthetic concentrations suggested for adult cephalopods ranged between 1% and 3% for EtOH and between 1.5% and 3.75% for MgCl_2_ depending on the species (reviewed in Fiorito et al., 2015). As no specific reference has been identified for *L. vulgaris*, we decided to investigate the effects of 2%, 2.5%, and 3% EtOH, as well as of 1%, 1.5%, and 1.8% MgCl_2_ (dissolved in 34 ppm artificial seawater). We did not assess the weight of the embryos. The whole procedure was video recorded, and the sequences were used to extract parameters.

### Parameter assessment

#### Heart rate

Like any other cephalopod, *Loligo vulgaris* possess three hearts, the systemic heart and two branchial hearts. Since the beating of three hearts is synchronized, the heart rate of only one heart was counted (typically the right branchial heart). During t0, the heart rate was recorded (as beats/minute) during minutes 1, 3, and 5, then a mean of the three values was calculated. During t1, the heart rate was recorded at minutes 6, 8, and 10, and a mean was then calculated. Finally, during t2, the heart rate was assessed at minutes 11, 13, and 15, and the mean was calculated.

#### Respiratory rate

Cephalopod respiration is a complex movement of the mantle, increases or decreases its size, the first one corresponding to an inspiration (Figures 3A,B) and the increase of pressure in the mantle (Figure 3B) triggering water expulsion through the funnel (expiration) (Bone et al., 1994). During t0, the contractions of the mantle combined with the funnel movements were counted during minutes 1, 3, and 5, then the mean of the three values was calculated. Similarly, during t1, respirations were counted during minutes 6, 8, and 10, and the mean value was calculated. During t2, respirations were counted at minutes 11, 13, and 15, and the mean was calculated.

#### Time until loss and to recovery of buoyancy “TL B”, “TR B”

Buoyancy is the capacity of the embryo to swim in a physiological manner and, if put in an unphysiological position, to return to a physiological one. Buoyancy should be present as proof of good health (Sherrill et al., 2000; Moltschaniwskyj et al., 2007) and thus a core criterion for embryos inclusion in the study. During t0, buoyancy was verified and defined as present or absent. During t1, the time until loss of buoyancy was measured in seconds once the embryo went from a present to an absent state. During t2, embryos were stimulated every 10 s by a gentle touch to the mantle, head, tentacles, and arms, and the time to recovery of buoyancy was recorded.

#### Time until loss and to recovery of chromatophore activity “TL CR”, “TR CR”

Chromatophores are pigmented sacs connected to radial muscles, and each contraction and relaxation of these muscles trigger the pigmented sac to increase or decrease its size, respectively (Mathger and Hanlon, 2007). During t0, the chromatophores were observed, and three states were defined: a relaxed state, half-contracted state, and a contracted state (Figure 5A–C). During t1, the time until loss of chromatophore activity was defined as the time from immersion in the anesthetic bath to loss of chromatophore activity or until a relaxed state was observed and maintained. During t2, the time to recovery of the chromatophore activity was defined as the time from immersion in an anesthetic-free solution to the return of chromatophore pattern changes/change from a relaxed to contracted state.

#### Time until loss and to recovery of tentacles/arms response “TL T/A R″, “TR T/A R”

In cephalopods, such as octopus, a measure of the state of anesthesia is sucker adhesiveness (Sen and Tanrikul, 2009; Pagano et al., 2011). However, for smaller cephalopods, sucker adhesiveness is difficult to evaluate. Moreover, this is particularly difficult to assess in late-stage *L. vulgaris* embryos. Therefore, as an alternative proxy for the responsiveness of the embryo, we assessed tentacles/arms response following a gentle pinch. During t0, the tentacles/arms response was classified as absent or present. During t1, the time until loss of tentacles/arms response or the absence of it was measured. In t2, the time to recovery of tentacles/arms response or its presence was recorded.

#### Typical adverse response patterns

The typical adverse response pattern (TARP) corresponds to an excitatory phase of immersion in the anesthetic solution that in some circumstances may be particularly marked, including inking, escape reactions, increased or erratic ventilation, rapid switch in coloration or of the chromatophore tone, rapid jetting movements, convulsions, and defecation (Gleadall, 2013). Any observed adverse reactions were recorded during induction and recovery.

### Data analysis

Video recordings of *L. vulgaris* embryos at stages 27–28 (Figure 1B) under different treatments were obtained during experiments, and parameters were noted from video playback. Each video consisted of a 5-min control phase (t0), a 5-min anesthesia phase (t1), and a 5-min recovery phase (t3), as mentioned above.

Statistical analyses were performed using standard statistical functions in jamovi (https://www.jamovi.org/). For all experiments where two conditions are directly compared, we used a t-test (buoyancy, chromatophores activity, and tentacles response), while for the experiments with multiple conditions, we used ANOVA with a Tukey *post hoc* test (heart and respiratory rates). A paired samples t-test was used to determine if there was a significant difference between the means of the embryos anesthetized with EtOH and those anesthetized with MgCl_2_, for the following parameters: buoyancy, chromatophores activity, and tentacles response. The heart and respiratory rates were assessed using repeated measures ANOVA within factors; three conditions and three repeated measures with a Tukey *post hoc* test. All data are presented as means ± standard error of the mean (SE).

### Ethical statement

Ethical review and approval were not required for this study because only embryos were used, which do not fall under the Swiss Animal Protection Ordinance. However, we followed the principles stated in the Guidelines for the Care and Welfare of Cephalopods in Research (Fiorito et al., 2015). After the experiment, the embryos were humanely killed by an increased dose of EtOH every 5 min, starting with 1.5%, then 2.5% until a final concentration of 3.75% was reached, and death was confirmed by mechanical brain/head destruction.

## Results

During the experiments, we did not observe mortality at short term, induced from treatment in *L. vulgaris* embryos.

### Heart rate

**FIGURE 2:**
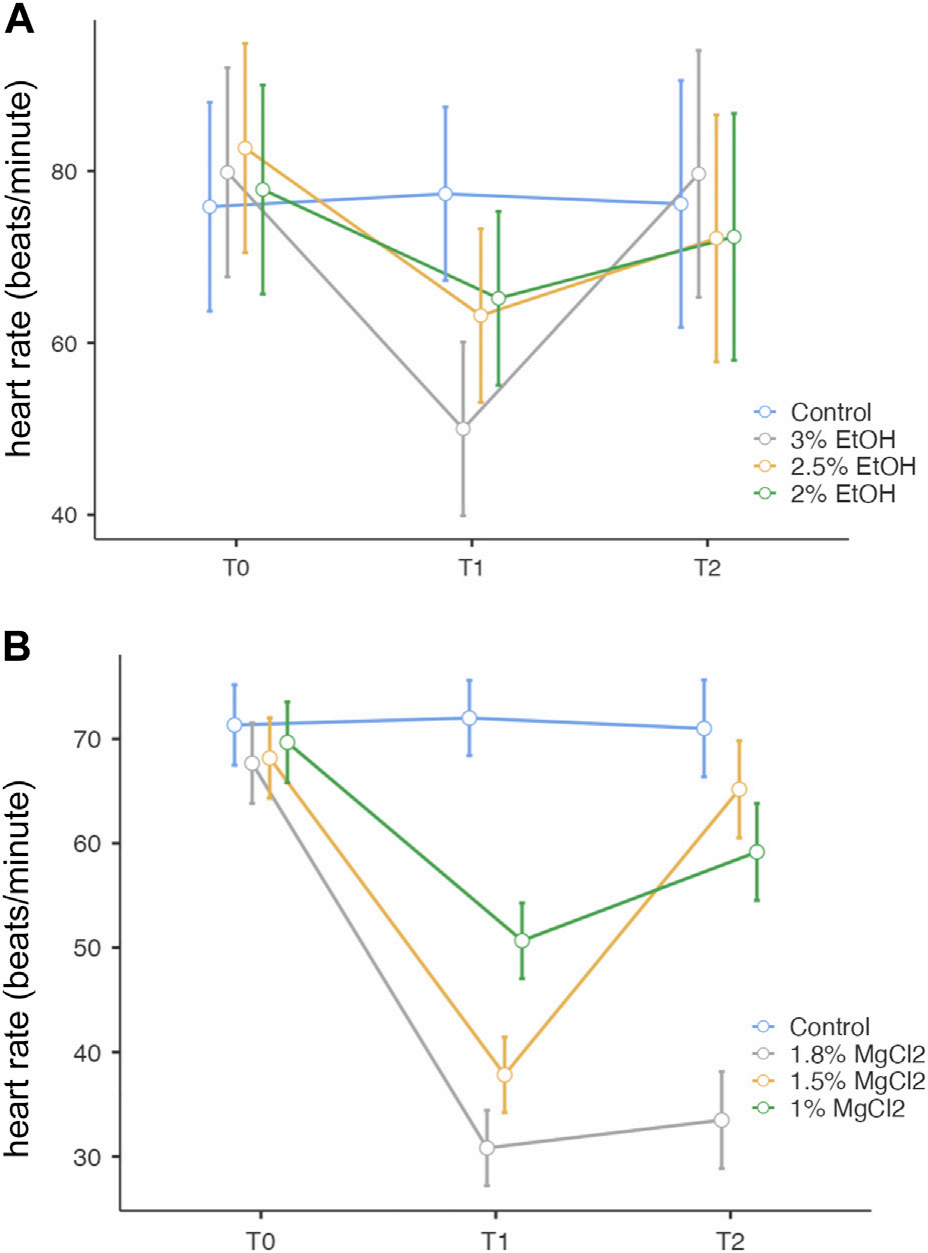
**(A)**. Heart rate (beats/minute) before, during, and after anesthesia for the three concentrations of EtOH (3%, 2.5%, and 2%), as well as the control group. The y-axis represents the heart rate in beats per minute, the x-axis displays the three phases of the experiment each lasting 5 min: T0, control phase; T1, sedation phase; and T2, recovery phase. **(B)** Heart rate (beats/minute) before, during, and after anesthesia for the three concentrations of MgCl_2_ (1.8%, 1.5%, and 1%), as well as the control group. The y-axis represents the heart rate in beats per minute, the x-axis displays the three phases of the experiment, each lasting 5 min: T0, control phase; T1, sedation phase; and T2, recovery phase.

During the control/baseline phase (t0), the heart rate was comparable in all experimental groups: mean ± SE, 79.04 ± 2.92 beats/minute (EtOH group) and 72.07 ± 5.06 beats/minute (MgCl_2_ group; Figure 2 A and B). It is noteworthy to report that we recorded the lowest observed rate at t0 of 67.67 beats/minute (±3.86) in animals assigned to the 1.8% MgCl_2_ group, and the highest values noted reached 82.67 beats/minute (±5.83; 2% EtOH group; see Table 1).

**TABLE 1:** Heart rate (beats/minute) of *L. vulgaris* embryos exposed to different EtOH and MgCl_2_ concentrations and the controls (no anesthetic solution). Measures are summarized (mean, SE, and 95% confidence interval) at the three different phases: baseline, t0; anesthesia, t1; and recovery, t2.

EtOH					
Concentrations	Time	Mean	SE	95% confidence interval	
				Lower	Upper
Control	T0	75.8	5.83	63.7	88.0
	T1	77.3	4.84	67.2	87.4
	T2	76.2	6.88	61.8	90.5
3% EtOH	T0	79.8	5.83	67.7	92.0
	T1	50.0	4.84	39.9	60.1
	T2	79.7	6.88	65.3	94.0
2.5% EtOH	T0	82.7	5.83	70.5	94.8
	T1	63.2	4.84	53.1	73.3
	T2	72.2	6.88	57.8	86.5
2% EtOH	T0	77.8	5.83	65.7	90.0
	T1	65.2	4.84	55.1	75.3
	T2	72.3	6.88	58.0	86.7
MgCl_2_					
Control	T0	71.3	3.86	63.3	79.4
	T1	72.0	3.61	64.5	79.5
	T2	71.0	4.65	61.3	80.7
1.8% MgCl_2_	T0	67.7	3.86	59.6	75.7
	T1	30.8	3.61	23.3	38.4
	T2	33.5	4.65	23.8	43.2
1.5% MgCl_2_	T0	68.2	3.86	60.1	76.2
	T1	37.8	3.61	30.3	45.4
	T2	65.2	4.65	55.5	74.9
1% MgCl_2_	T0	69.7	3.86	61.6	77.7
	T1	50.7	3.61	43.1	58.2
	T2	59.2	4.65	49.5	68.9

During the anesthesia phase (t1), the lowest heart rate was reached under 1.8% MgCl_2_ at 30.83 beats/minute (±3.61), followed by the rate noted under 1.5% MgCl_2_ (37.83 beats/minute) and 1% MgCl_2_ (50.67 beats/minute). The lowest heart rate for the EtOH group was triggered by the 3% EtOH concentration with 50.00 beats/minute (±4.84) followed by the 2.5% EtOH group with 63.17 beats/minute, and finally the 2% EtOH group recorded the highest heart rate for both groups with 65.17 beats/minute (Figure 2 A, B, and Tables 1).

During the recovery phase (t2), animals returned to values comparable to the baseline with the highest heart rates observed for the EtOH group with 79.67 beats/minute (±6.88), 72.33 beats/minute, and 72.17 beats/minute for the 3%, 2%, and 2.5% concentrations, respectively. For the MgCl_2_ group, the highest values of the heart rate were observed in the 1.5% concentration condition (65.17 ± 4.65 beats/minute), followed by the 1% concentration condition with 59.17 beats/minute and the 1.8% concentration condition with 33.50 beats/minute (see Table 1).

Overall, no significant differences emerged among the groups (*p* = 1, 3% EtOH and 2% EtOH; *p* = 0.999 for the 2.5% EtOH group). By comparing the heart rates between t0 and t1, significant differences resulted (3% EtOH and 2.5% EtOH, *p* < 0.001 and *p* = 0.003, respectively), confirming the heart rate depression during anesthesia. However, no variations existed between t0 and t1 for the 2% EtOH group, confirming that the embryos were too lightly anesthetized, as were those in the control group with *p* = 0.113 and *p* = 1, respectively. During the recovery phase, significant variations were found only for the 3% EtOH group (*p* < 0.001) (see Supplementary Table S1).

Overall, no significant differences were observed during t0 (baseline) between different treatments (*p* > 0.05). However, significant differences existed when comparing before anesthesia (t0) and during anesthesia (t1; *p* < 0.001 for 1.8% and 1.5%, *p* = 0.002 for 1% MgCl_2_) except for the control group (*p* = 1), thus confirming the heart rate depression induced by anesthesia. When comparing the heart rate between the anesthesia (t1) and recovery (t2) phases, no significant differences were observed for animals exposed to 1.8% MgCl_2_ since the animals never recovered and the 1% appeared to be only light sedated. *p* < 0.001 for the 1.5% MgCl_2_ proved to be the more satisfying concentration (Supplementary Table S1).

### Respiratory rate

**FIGURE 3:**
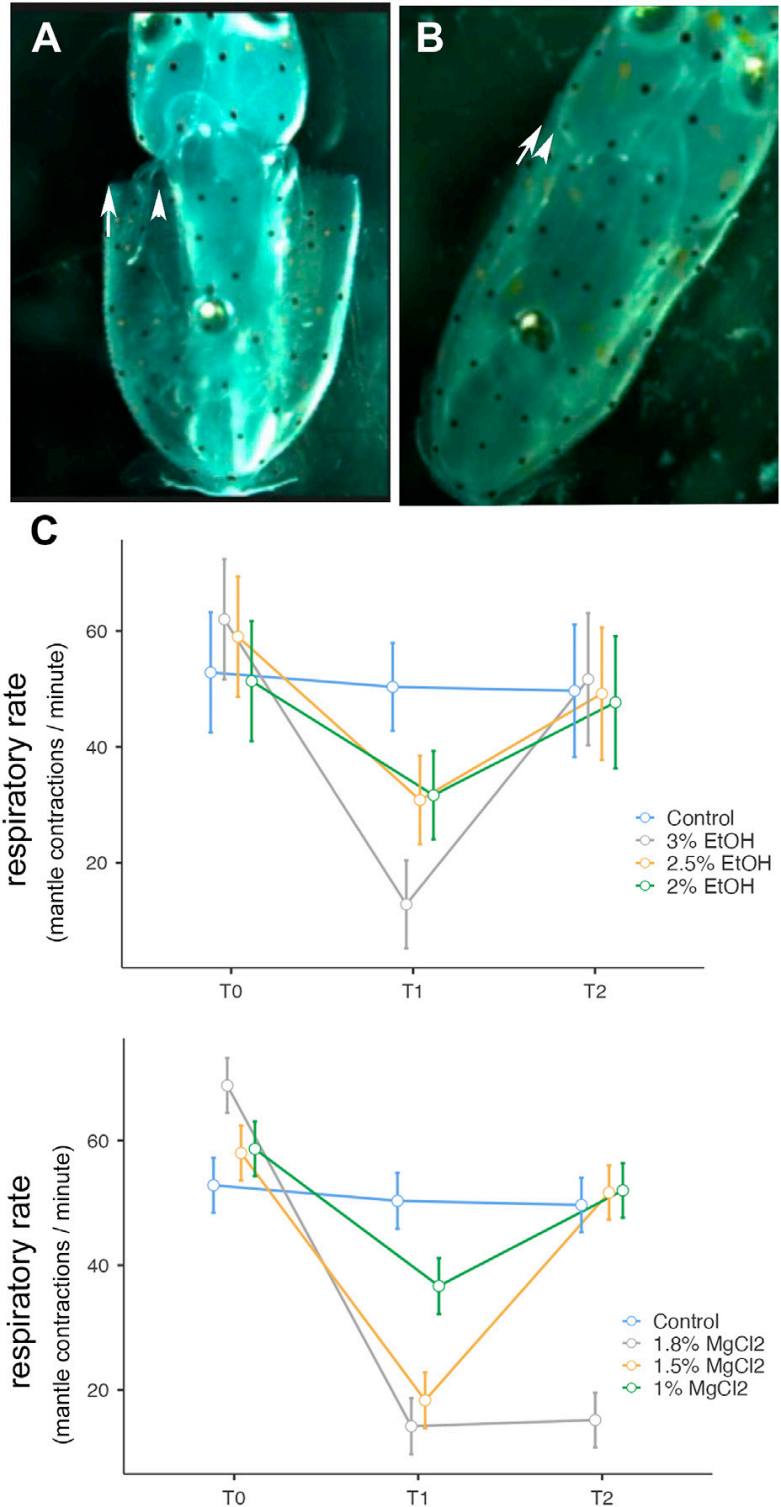
**(A)** Expansion of the mantle. **(B)** Contraction of the mantle. Both **(A)** and **(B)** correspond to one respiration. **(C)** Respiratory rate (mantle contractions/minute) before, during, and after anesthesia for the three concentrations of EtOH (3%, 2.5%, and 2%) and MgCl_2_ (1.8%, 1.5%, and 1%), as well as the control group. The y-axis represents the respiratory rate, the x-axis displays the three phases of the experiment, each lasting 5 min; T0, control phase; T1, sedation phase; and T2, recovery phase.

Table 2 summarizes the respiratory rate observed in *L. vulgaris* embryos in different phases and conditions of the experiment. During the t0 phase, the average respiratory rate ranged between 56.29 ± 5.50 and 63.40 ± 6.70 mantle contractions per minute (EtOH and MgCl_2_ groups, respectively (Figure 3C), with a peak of 68.83 ± 3.86 mantle contractions per minute (mcm), and the lowest values recorded were 51.33 mantle contractions per minute. During anesthesia phase (t1), the respiratory rate was reduced to 12.83 ± 3.64 mcm (3% EtOH) and 14.20 ± 4.49 mcm (1.8% MgCl_2_; Figure 3 C; Table 2). The second lowest respiratory rates were recorded with 1.5% MgCl_2_ concentration (18.33) followed by 2.5% EtOH and 2% EtOH groups (30.83 and 31.70 mantle contractions per minute, respectively). The less depressed respiratory rate in squid embryos was observed with the 1% MgCl_2_ concentration, reaching 36.70 mantle contractions per minute (50.33 mantle contractions per minute for the control group; Table 2). During recovery (t2), the respiratory rate returned to high values of 52.0 ± 4.37 mcm (1% MgCl_2_), 51.67 mcm (1.5% MgCl_2_), and 51.68 (3% EtOH group) mantle contractions per minute; 49.17 mcm (2.5% EtOH) and 47.70 mcm for *L. vulgaris* embryos exposed to 2% EtOH concentrations. We registered 49.67 mantle contractions per minute in the control group (Table 2).

**TABLE 2:** Respiratory rate (mantle contractions/minute) of *L. vulgaris* embryos exposed to different EtOH and MgCl_2_ concentrations and the controls (no anesthetic solution). Measures are summarized (mean, SE, and 95% confidence interval) at the three different phases: baseline, t0; anesthesia, t1; and recovery, t2.

EtOH					
Concentrations	Time	Mean	SE	95% Confidence interval	
				Lower	Upper
Control	T0	52.8	4.97	42.46	63.2
	T1	50.3	3.64	42.74	57.9
	T2	49.7	5.47	38.25	61.1
3% EtOH	T0	62.0	4.97	51.63	72.4
	T1	12.8	3.64	5.24	20.4
	T2	51.7	5.47	40.25	63.1
2.5% EtOH	T0	59.0	4.97	48.63	69.4
	T1	30.8	3.64	23.24	38.4
	T2	49.2	5.47	37.75	60.6
2% EtOH	T0	51.3	4.97	40.96	61.7
	T1	31.7	3.64	24.07	39.3
	T2	47.7	5.47	36.25	59.1
MgCl_2_					
Control	T0	52.8	4.39	43.68	62.0
	T1	50.3	4.49	40.96	59.7
	T2	49.7	4.37	40.56	58.8
1.8% MgCl_2_	T0	68.8	4.39	59.68	78.0
	T1	14.2	4.49	4.80	23.5
	T2	15.2	4.37	6.06	24.3
1.5% MgCl_2_	T0	58.0	4.39	48.84	67.2
	T1	18.3	4.49	8.96	27.7
	T2	51.7	4.37	42.56	60.8
1% MgCl_2_	T0	58.7	4.39	49.51	67.8
	T1	36.7	4.49	27.30	46.0
	T2	52.0	4.37	42.89	61.1

No differences were observed during t0 (baseline; see Supplementary Table S2). However substantial differences were noted when t0 (before anesthesia) and t1 (after anesthesia) phases were compared (*p* < 0.001, 1.8%, and 1.5% MgCl_2_ concentration groups). No differences emerged when embryos exposed to 1% MgCl_2_ were considered (*p* = 0.079; Supplementary Table S2). In addition, when baseline (t0) and the recovery (t2) phases were considered, we did not find significant differences except for the 1.8% MgCl_2_ group. Comparison between the anesthesia phase (t1) and the recovery (t2) revealed significant differences for the 1.5% (*p* < 0.001) and 1% (*p* = 0.078) MgCl_2_ groups, showing recovery which appears to be more efficient when the 1.8% concentration was considered (Supplementary Table S2).

### Time until loss of buoyancy and time to recovery of buoyancy

**FIGURE 4:**
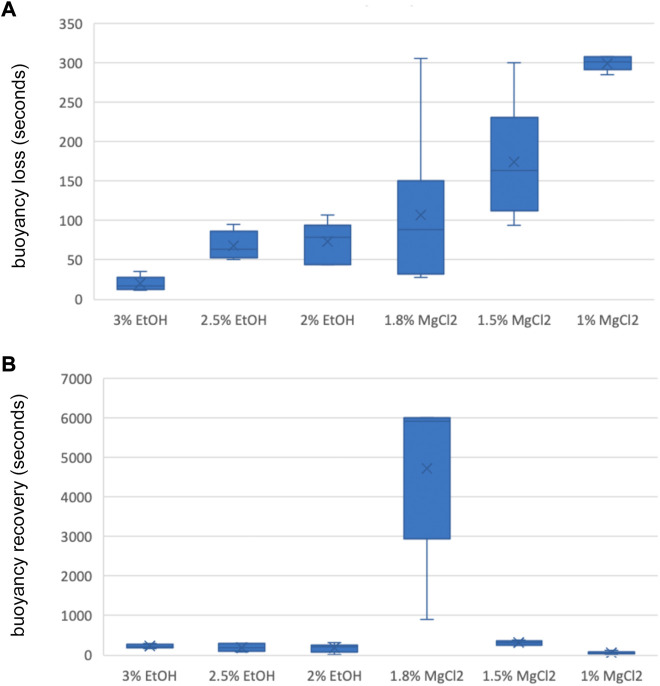
**(A)** Box and whiskers plot with the time until loss of buoyancy response in seconds. The y-axis represents the seconds until loss of buoyancy and the x-axis displays the three EtOH and MgCl_2_ concentrations. The upper and lower limits show the highest and lowest times, respectively, until the loss of buoyancy, and the mid-point shows the mean. **(B)** Box and whiskers plot with the time to recovery of buoyancy response in seconds. The y-axis represents the seconds to the recovery of buoyancy and the x-axis displays the three EtOH and MgCl_2_ concentrations. The upper and lower limits show the highest and lowest times, respectively, until recovery of buoyancy, and the mid-point shows the mean.

Time until loss of buoyancy “TL B″ was shorter on average when using EtOH (54 ± 8.29 s) compared to values recorded with MgCl_2_ (194 ± 52.61 s; Figure 4 A and B). The faster time until loss of buoyancy was reached when embryos were exposed to 3% EtOH concentration (19.83 ± 3.66 s; Table 3), followed by the 2.5% EtOH concentration and the 2% EtOH concentration (68.33 ± 7.17, and 73.83 ± 10.44 s, respectively). For the MgCl_2_ group, the TL B took longer (TL B: 107.50 ± 41.60 s, 1.8% MgCl_2_; 174.50 ± 30.09 s, 1.5% MgCl_2_; 299.50 ± 3.69 s, 1% MgCl_2_; Table 3). During the recovery phase after anesthesia (t2), the average time to recovery buoyancy “TR B″ was 198.83 ± 33.25 s for the EtOH group but required 1700.06 ± 1185.52 s for the MgCl_2_ group, thus suggesting a marked interindividual variability in this case (Table 3). The highest TR B value was observed for embryos exposed to 1.8% MgCl_2_ (4721.67 ± 855.4 s), followed by animals of the 1.5% MgCl_2_ concentration (314 ± 21.60 s), then by the 3% EtOH group (223.33 ± 20.00 s) and the 1.5% and 2% EtOH groups (194.67 ± 42.70 and 178.50 ± 44.40 s, respectively). The faster TR B was obtained with 1% MgCl_2_ concentration at 64.50 ± 12.60 s (Table 3).

**TABLE 3:** Time until loss of buoyancy in seconds (TL B) and time to recovery of buoyancy in seconds (TR B) of *L. vulgaris* embryos exposed to different EtOH and MgCl_2_ concentrations and the controls (no anesthetic solution). Measures are summarized (mean, median, SD, and SE).

	N	Mean	Median	SD	SE
TL B 3% EtOH	6	19.8	16.5	8.98	3.66
TL B 2.5% EtOH	6	68.3	63.5	17.57	7.17
TL B 2% EtOH	6	73.8	79.0	25.56	10.44
TL B 1.8% MgCl_2_	6	107.5	89.0	101.90	41.60
TL B 15% MgCl_2_	6	174.5	163.5	73.69	30.09
TL B 1% MgCl_2_	6	299.5	301.5	9.05	3.69
	**N**	**Mean**	**Median**	**SD**	**SE**
TR B 3% EtOH	6	223.3	216.5	49.1	20.0
TR B 2.5% EtOH	6	194.7	194.0	104.5	42.7
TR B 2% EtOH	6	178.5	198.0	108.7	44.4
TR B 1.8% MgCl_2_	6	4721.7	5910.0	2095.2	855.4
TR B 1.5% MgCl_2_	6	314.0	320.0	53.0	21.6
TR B 1% MgCl_2_	6	64.5	70.0	30.9	12.6

No significant differences were obtained for the time until loss of buoyancy among all three EtOH concentrations and the 1.8% MgCl_2_ concentration (Supplementary Table S3). However, significant differences were found in comparing EtOH concentrations and the 1.5% and 1% MgCl_2_ groups (Supplementary Table S3). Substantial differences existed among groups; the 3% EtOH concentration was significant when compared to the baseline, while the 1.8% and 1.5% MgCl_2_ concentrations had no significant differences (Supplementary Table S3).

Concerning the time to recover buoyancy, no significant differences existed between the EtOH concentrations and the 1.5% MgCl_2_ group (Supplementary Table S3). Among the EtOH concentrations, no significant difference emerged for time to recover buoyancy, in contrast to the case of the MgCl_2_ group (all significant; Supplementary Table S3).

### Time until loss of chromatophore activity and time to recovery of chromatophores activity

**FIGURE 5:**
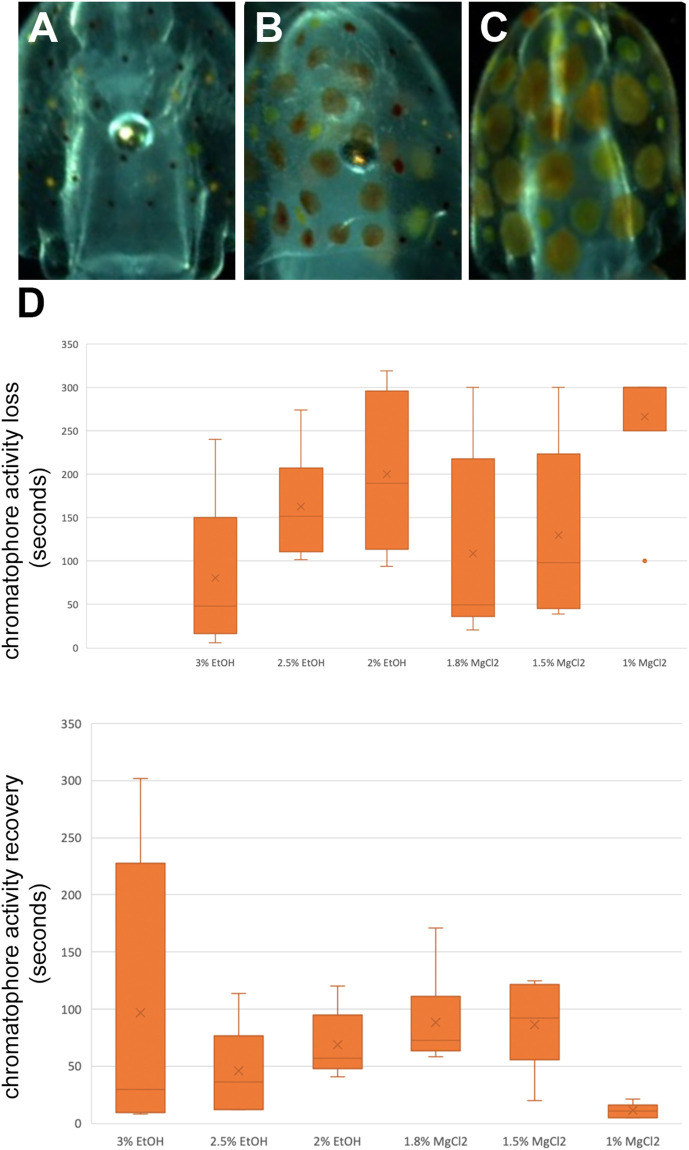
**(A)** Contracted chromatophores corresponding to muscle relaxation. **(B)** A half-contracted chromatophores. **(C)** Completely relaxed chromatophores corresponding to full muscle contraction. **(D)** Box and whiskers plot with the time until the loss or to recovery of chromatophores activity in seconds. The y-axis represents the seconds until the loss or to recovery of chromatophores and the x-axis displays the three EtOH and MgCl_2_ concentrations. The upper and lower limits show the highest and lowest times, respectively, until loss or to recovery of chromatophore activity, and the mid-point shows the mean.

Time until loss of chromatophore activity “TL CR” was faster on average with EtOH (147.89 ± 14.45 s) than with MgCl_2_ (168.44 ± 15.26 s; Figure 5D). The faster onset was with the 3% EtOH concentration at 80.50 ± 36.40 s, followed by the 1.8% MgCl_2_ group (108.50 ± 45.70 s; Table 4). The loss of chromatophore activity was reached in 130.17 ± 41.10 s when squid embryos were exposed to 1.5% MgCl_2_ concentration and in 163.17 ± 26.30 s when the animals were exposed to 2.5% EtOH concentration (Table 4). On the other hand, TL CR required more than 200 s when the squid embryos were tested with 2% EtOH (200 ± 36.70 s) and 1% MgCl_2_ (266.67 ± 33.33 s; Table 4).

**TABLE 4:** Time until loss of chromatophore tone in seconds (TL CR) and time to recovery of chromatophore activity in seconds (TR CR) of *L. vulgaris* embryos exposed to different EtOH and MgCl_2_ concentrations and the controls (no anesthetic solution). Measures are summarized (mean, median, SD, and SE).

	N	Mean	Median	SD	SE
TL CR 3% EtOH	6	80.5	48.5	89.1	36.4
TL CR 2.5% EtOH	6	163.2	152.0	64.4	26.3
TL CR 2% EtOH	6	200.0	189.5	89.8	36.7
TL CR 1.8% MgCl_2_	6	108.5	49.5	111.8	45.7
TL CR 1.5% MgCl_2_	6	130.2	98.0	100.6	41.1
TL CR 1% MgCl_2_	6	266.7	300.0	81.6	33.3
	**N**	**Mean**	**Median**	**SD**	**SE**
TR CR 3% EtOH	6	97.0	29.5	125.34	51.17
TR CR 2.5% EtOH	6	45.8	36.5	39.51	16.13
TR CR 2% EtOH	6	68.5	57.0	29.46	12.03
TR CR 1.8% MgCl_2_	6	88.3	72.5	41.97	17.13
TR CR 15% MgCl_2_	6	86.2	92.0	38.88	15.87
TR CR 1% MgCl_2_	6	11.2	11.0	6.05	2.47

Recovery of chromatophore tone was quite fast with both MgCl_2_ and EtOH (61.89 ± 19.91; 70.44 ± 52.69 s, respectively). The fastest recovery was recorded when the lowest MgCl_2_ concentration (1%) was used (11.17 ± 2.47 s) and the 2.5% EtOH group (45.83 ± 16.13 s; Table 4). More time was required when animals were exposed to the 2% EtOH concentration (68.50 ± 12.03 s; Table 4). On the other hand, 3% EtOH concentration and 1.8% MgCl_2_ concentration resulted in a longer time to recovery of chromatophore tone (97 ± 51.17; 88.33 ± 17.13 s, respectively; Table 4). It is interesting to note that embryos that exhibited half-contracted or relaxed chromatophore states at the baseline performed a similar pattern during the whole immersion in EtOH until the change to fresh artificial seawater.

There were no significant differences in the time to recovery of chromatophore activity among the three concentrations of EtOH and the three concentrations of MgCl_2_. However, significant differences were observed between the 1.8% and 1% MgCl_2_ and between 1.5% and 1% MgCl_2_ (Supplementary Table S4) groups.

### Time until loss and recovery of tentacle response

**FIGURE 6:**
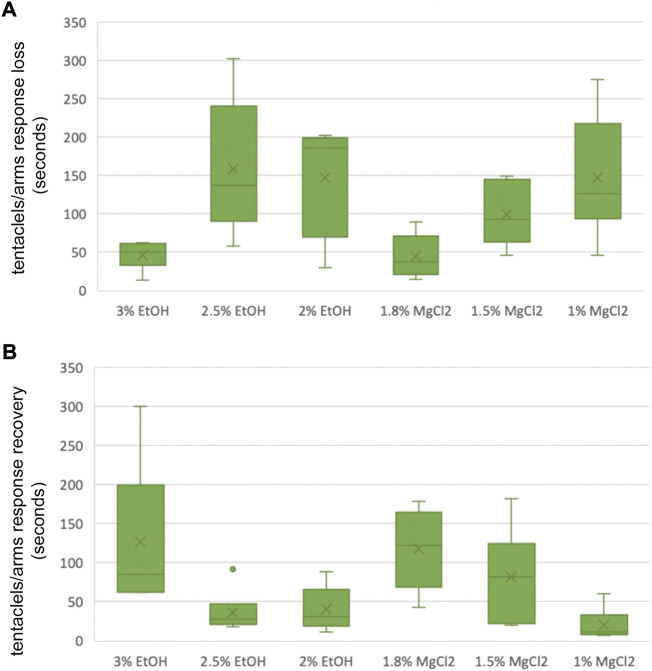
**(A)** Box and whiskers plot with the time until loss of tentacles/arms response in seconds. The y-axis represents the seconds until loss of tentacles/arms response and the x-axis displays the three EtOH and MgCl_2_ concentrations. The upper and lower limits show the highest and the lowest times, respectively, until the loss of tentacles/arms response, and the mid-point shows the mean. **(B)** Box and whiskers plot with the time to recovery of tentacles/arms response in seconds. The y-axis represents the seconds to recovery of tentacles/arms response and the x-axis displays the three EtOH and MgCl_2_ concentrations. The upper and lower limits show the highest and the lowest times, respectively, until the recovery of tentacles/arms response, and the mid-point shows the mean.

Time until loss of the tentacles/arms response “TL T/A R” was quicker on average for the MgCl_2_ concentrations with 96.94 ± 25.26 s and 117.83 ± 37.54 s for the EtOH concentrations (Figure 6 A and B). The faster TL T/AR was achieved by the 1.8% MgCl_2_ concentration with 44.83 ± 11.70 s, followed by 46.33 ± 7.43 s for the 3% EtOH concentration. The second fastest TL T/A R resulted in conditions where animals were exposed to 1.5% MgCl_2_ concentration (99 ± 16.54 s); this was followed by TL T/A R in animals exposed to 2% MgCl_2_ concentration (147.67 ± 29.66 s) and 2.5% EtOH (159.51 ± 36.81; Table 5). The slower TL T/A R was observed for squid embryos exposed to 1% MgCl_2_ concentrations (266.70 ± 33.33 s; Table 5). Time to the recovery of tentacles/arms response “TR T/A R″ was on average quicker for the EtOH (67.94 ± 37.85 s) than was for the MgCl_2_ (73.28 ± 28.37 s) group. We observed the quickest recovery in the 1% MgCl_2_ group, followed by the groups exposed to 2.5% EtOH and 2% EtOH (20.17 ± 8.35; 36.67 ± 11.30; 40.33 ± 11.55 s, respectively; Table 5). The longest TR T/A R was obtained for the 3% EtOH concentration (126.83 ± 38.19 s) and 1.8% MgCl_2_ (117.33 ± 21.75 s; Supplementary Table S5) groups.

**TABLE 5:** Time until loss of tentacles/arms response in seconds (TL T/A R) and time to recovery of tentacles/arms response in seconds (TR T/A R) of *L. vulgaris* embryos exposed to different EtOH and MgCl_2_ concentrations and the controls (no anesthetic solution). Measures are summarized (mean, median, SD, and SE).

	N	Mean	Median	SD	SE
TL T/A R 3% EtOH	6	46.3	50.0	18.2	7.43
TL T/A R 2.5% EtOH	6	159.5	137.5	90.2	36.81
TL T/A R 2% EtOH	6	147.7	186.0	72.7	29.66
TL T/A R 1.8% MgCl_2_	6	44.8	38.0	28.7	11.70
TL T/A R 1.5% MgCl_2_	6	99.0	93.0	40.5	16.54
TL T/A R 1% MgCl_2_	6	266.7	300.0	81.6	33.3
	**N**	**Mean**	**Median**	**SD**	**SE**
TR T/A R 3% EtOH	6	126.8	85.5	93.5	38.19
TR T/A R 2.5% EtOH	6	36.7	27.5	27.7	11.30
TR T/A R 2% EtOH	6	40.3	31.5	28.3	11.55
TR T/A R 1.8% MgCl_2_	6	117.3	122.0	53.3	21.75
TR T/A R 15% MgCl_2_	6	82.3	82.0	61.1	24.94
TR T/A R 1% MgCl_2_	6	20.2	11.0	20.5	8.35

### Typical adverse response pattern

No TARP reactions (as defined by Gleadall, 2013) were observed throughout the course of the study.

### Follow-up of the embryos after the anesthesia protocol

The embryos were monitored for 48 h after anesthesia to assess their vitality and any other adverse effects. Our data show that embryos exposed to MgCl_2_ can be divided into two groups: the first group are the embryos exposed to 1.8% concentration and the second group are those anesthetized with the 1.5% and 1% MgCl_2_ concentrations. None of the embryos exposed to 1.8% MgCl_2_ survived after 6 h. Besides buoyancy, all the other parameters considerably returned to values similar to the baseline; the heart and respiratory rates took between 40 min and 1 h to normalize. The chromatophore tone and tentacles/arms response also recovered relatively quickly within 5 min. However, the quickest recovery for the buoyancy was obtained after 2 h 46 min for one embryo and did not occur for the remaining embryos. On the other hand, 8 out of 12 embryos survived 24 h with a decreased heart rate and respiratory rate and only four survived 48 h. In any case, death appeared to be linked to respiratory depression followed by poor oxygenation.

The short life span can be explained by the type of anesthetic but also the fact that embryos had lost their external yolk, leaving them with only their internal yolk to feed them. For the EtOH group, for each concentration, only one embryo was kept for this follow-up. The three embryos those survived 24 h with a proper heart and respiratory rate, buoyancy, chromatophore, and tentacles/arms response were still present. After 48 h, the embryos were still present with a heart rate, but with a lower rate than the baseline, and the respiration was depressed with partial buoyancy.

## Discussion

### Anesthetic substances

The large diversity of cephalopods, with over 800 species, makes it difficult to standardize the use and dosage of anesthetic solutions. For research purposes, only a few species are currently being studied. However, these species have diverged, adapting to rather different lifestyles and differing in body sizes. While it is challenging to anesthetize different species, it is important to establish some common approaches and standardize the evaluation of anesthetic quality in these animals. In our study, the parameters proposed by Pagano et al. (2011) and Andrews et al. (2013) were investigated as much as the size and physiology of the *Loligo* embryos allowed.

The concentrations of EtOH and MgCl_2_ used in the present study were based on ranges previously utilized for adult animals (reviewed in Fiorito et al., 2015). Depending on the species and body weight, concentrations ranging from 1 to 3% were suggested for EtOH and from 1.5% to 3.75% for MgCl_2_. Therefore, in our study the concentrations for the EtOH solution tested were 2%, 2.5%, and 3% and were 1%, 1.5% and 1.8% for MgCl_2_ in seawater. It seems likely that the body size and age of the animal may affect the efficiency of a given anesthetic. In our study, the comparison of these substances revealed that for the heart rate and respiratory rate, EtOH had a faster onset and recovery than did MgCl_2_, confirming previous observations reported for adult squids. Indeed, a recent study found that EtOH is less depressive and easier to recover than MgCl_2_ for octopus and cuttlefish (Abbo et al., 2021). Nevertheless, the return of the chromatophore and tentacle/arm responses was faster for the MgCl_2_ groups than the EtOH groups.

The mechanisms of MgCl_2_ and EtOH anesthetic functions in cephalopods is still largely unknown. A recent study in *Sepia pharaonis* showed that MgCl_2_ enhances the amount of the inhibitory neurotransmitters glycine and tyrosine while reducing the level of dopamine (Yang et al., 2020). In contrast to EtOH, MgCl_2_ has a prolonged activity, being therefore potentially more suitable for longer procedures. In addition, MgCl_2_ has been reported to have not only immobilizing properties but also analgesic ones, by acting as a neurotransmitter release blocker and having local anesthetic properties (Butler-Struben et al., 2018). However, the mode of action and physiological mechanism of MgCl_2_ as an anesthetic have not been fully elucidated and remain to be explored. It has been studied extensively in mice (Wong et al., 1997; Sharko and Hodge, 2008). According to Wong et al. (1997), the use of EtOH resembles the use of volatile anesthetics such as desflurane, both depressing the muscle stretch reflex or monosynaptic reflex while having a quick onset and offset of action as well as an age-related rise of anesthetic potency. The younger the animal, the higher the required dosage; the latter is along the lines of the higher dosage used in our experiment. In addition, EtOH has a hypnotic and sedative effect modulated through metabotropic glutamate receptors requiring GABA_A_ and NMDA receptors. Even though EtOH analgesic effects are not known in cephalopods, it is notorious to provoke a possible status of loss of consciousness in humans, followed in some cases of amnesia and analgesic effects (Tamerin et al., 1971; Thompson et al., 2017), but state dependency (Goodwin et al., 1969; Bettinger and McIntire, 2004; Rezayof et al., 2008) has been widely reported and may be the cause of such effects.

In the future, it will be possible to compare different concentrations, combinations of substances, or other anesthetics (e.g., isoflurane; Polese et al., 2014). In the present study, embryos were anesthetized by immersion, which is a minimally invasive approach, largely utilized in fish, cephalopods, and other aquatic organisms. Other administration methodologies exist, such as subcutaneous or intravenous injections, and might be explored for future studies, with appropriate care in terms of monitoring stress and severity degrees.

The heart rate and respiratory rate are basic vital parameters that can be easily recorded during anesthesia for cephalopods and, particularly for, *Loligo vulgaris* embryos, and no reference values are available for these vital parameters in the literature. Therefore, obtaining baseline data before and after anesthesia was one of our main goals.

### Heart and respiratory rate

During the baseline phase, the heart rate and respiratory rate were recorded, and they were consistent among all embryos. During anesthesia, the heart and respiratory rates were depressed for both anesthetics, however, important differences among embryos were observed, corroborating the idea that individual susceptibility to anesthetic concentration is present (Butler-Struben et al., 2018). The best EtOH concentration remained 3%, in comparison to the 2.5% and 2% EtOH concentrations (but mortality at the end of the experiment should be carefully considered). For the latter two, the embryos were lightly anesthetized, and the decrease in the heart and respiratory rates showed no significant differences between the three phases (Figure 2 and 3, t1–t2). EtOH 3% concentration displayed the quickest return to the initial baseline: within 1 min. The respiration rate at the 3% EtOH concentration resulted as the lowest of all concentrations and returned similarly to baseline within 3 min, similar to the most optimal MgCl_2_ concentration. Comparing the heart and respiratory rate parameters, the optimal MgCl_2_ solution was the 1.5% concentration; the heart rate obtained after anesthesia was equivalent to the 1.8% MgCl_2_ concentration but lower than the 3% EtOH concentration. The return to baseline was obtained within 6 min instead of nearly 40 min to 1 h for the 1.8% MgCl_2_ concentration. The respiratory rate attained with the 1.5% concentration was the third lowest rate after the 1.8% MgCl_2_ concentration and the 3% EtOH concentration. Concerning the recovery, it was reached within the observed 5 min.

### Tentacles/arms response

In mammals, the return to a responsive and conscious state following general anesthesia is typically evaluated by looking at eye reflexes, jaw tone, and occurrence of spontaneous movements. In cephalopods, tentacle adhesiveness as well as eye reflexes have been proposed as signs of recovery (Sen and Tanrikul, 2009; Pagano et al., 2011). Due to the smaller size of the embryos used in this study, these parameters would have been difficult to assess. Therefore, the tentacles/arms response to pinch was used instead. This response was lost quickly on average with MgCl_2_, especially at the 1.8% concentration, than with EtOH, but recovery was never reached for this concentration. Nevertheless, lower dosages of 1% MgCl_2_ attained the quickest recovery time. Corroborating the mechanism of action of MgCl_2_ (Yang et al., 2020), indeed MgCl_2_ not only releases inhibitory neurotransmitters but at the same time also reduces the release of excitatory neurotransmitters, and therefore lower concentrations could have less depressive effects.

### Chromatophore tone

Cephalopods possess unique features such as chromatophore activity, allowing adaptation to environmental conditions. Indeed, while submerging the embryos in both anesthetic agents, the chromatophore reacted until a complete contraction (paling). Both MgCl_2_ and EtOH showed a similar effect and no differences existed among the concentrations for the time until the loss and recovery of function. The faster TL CR was obtained with 3% EtOH, with the longer TR CR being registered for the 1.8% MgCl_2_ concentration, possibly indicating that at higher concentrations, MgCl_2_ is a better muscle relaxant than EtOH.

### Buoyancy

Buoyancy is another physiological mechanism that healthy squids should display. According to previously published guidelines for aquatic species, anesthesia is considered “effective” if it acts within 3 min of immersion in an anesthetic bath, while recovery should occur within 5 min after returning to an anesthetic-free solution (Marking and Meyer, 1985). Both anesthetics were quite efficient in inducing loss of buoyancy, no significant differences existed for the three EtOH solutions and 1.8% MgCl_2_. Regarding the recovery of function, it was quicker with EtOH than with MgCl_2_. Since the 1.8% MgCl_2_ concentration took longer than 300 s to recover, it indicates a non-recovery of function, and therefore according to the definition of Marking and Meyer (1985), does not fulfill the efficiency criteria. In addition, no significant differences existed between the 3% EtOH, 2.5% EtOH, and 1.5% MgCl_2_ concentrations for the time to recovery of buoyancy.

### TARP

Any adverse response pattern should be documented for embryos. According to Gleadall (2013), there are different adverse responses for embryos, such as inking, defecating, or rapid changes of chromatophore patterns. Immersion in EtOH and MgCl_2_ solutions was well tolerated by embryos of the common squid *Lolig*o *vulgaris*, and no adverse effects at the tested concentrations were observed, corroborating previous reports (Lange and Hartline, 1974; Garcia-Franco, 1992; Ikeda et al., 2009; Mooney et al., 2010; Estefanell et al., 2011).

## Conclusion

Following various authors, cephalopods react to anesthesia by changes in the respiratory rate, which appear depressed with the magnitude, depending on the concentration. Similar results were obtained in our study in squid embryos since their respiratory rates went down with differences in depth, depending on the concentrations used. Furthermore, the loss of body color or chromatophore relaxation are the other indicators of the depth of anesthesia. According to the substances used, adults look paler or achromatic. Indeed, for the embryos, this was also the case; their chromatophores relaxed, not allowing the pigmentation to show. Sucking intensity is the only parameter that could not be measured in embryos in this study due to their relative size. Instead, pinching of arms and tentacles was used.

We found that both substances had similar effects on the heart and respiratory rates after immersion, as well as on chromatophores activity, tentacles/arms response, and buoyancy. The main differences between EtOH and MgCl_2_ appeared during the recovery. Buoyancy was on average more quickly restored following immersion in EtOH than it did in MgCl_2_. For the 1.8% MgCl_2_ concentration, buoyancy was not regained, indicating that the safety for MgCl_2_ at this concentration cannot be guaranteed. On the other hand, no significant differences were observed for anesthetic recovery of the chromatophore and tentacle/arms responses.

It should be noted that considering the marked interindividual variability (also observed here), we cannot exclude that a fixed time frame of exposure to the anesthetic solution and recovery may be insufficient for all animals to attain a similar state for each concentration tested. Further studies may help in disclosing such a possibility.

Even though no adverse response pattern was observed during the experiment for both EtOH and MgCl_2_ at the investigated concentrations, EtOH seemed to be a more efficient substance for *Loligo vulgaris* embryos than did MgCl_2_ in circumstances here tested. Not only did embryos regain their heart and respiratory rates quickly but had also recovered physiological responsiveness more rapidly after the EtOH treatment.

Moreover, both substances might at increasing concentrations also be relevant for euthanasia.

The physiological parameters used here represent a critical attempt to standardize the monitoring of embryos, and together with other techniques such as electrophysiology recordings from the pallial nerves (Abbo et al., 2021) or H-NMR spectroscopy (Yang et al., 2020), could elucidate the mechanism of action of MgCl_2_ and EtOH, in particular, the reversible inhibitory activity of these substances. In future studies, further measures could be included, such as for instance, skin irritation (linked with exposure to EtOH) or corticosteroids production in response to exposure to anesthetics (Chancellor et al., 2021). Such studies are critical to gain deeper knowledge on the effects and safety of anesthetic agents in cephalopods, possibly highlighting differences among species and peculiarities in mechanisms of action.

## Data Availability

The original contributions presented in the study are included in the article/Supplementary Material; further inquiries can be directed to the corresponding author.
