# Supplementary material for: A pilot investigation of the efficacy and safety of magnesium chloride and ethanol as anesthetics in Loligo vulgaris embryos
Source: Front Physiol. 2022 Sep 14;13:968047. doi: 10.3389/fphys.2022.968047 (PMC9641376; doi:10.3389/fphys.2022.968047)

Supplementary Info to

**Pilot investigation of the efficacy and safety of Magnesium Chloride and Ethanol as anaesthetics in *Loligo vulgaris* embryos**

Marta Sprecher^1^, Simon G. Sprecher^1^ and Claudia Spadavecchia^2^*

^1^Department of Biology, University of Fribourg, Fribourg, Switzerland

^2^Department of Clinical Veterinary Medicine, Anaesthesiology and Pain Therapy Section, Vetsuisse Faculty, University of Bern, Switzerland

^*^ Correspondence: Marta Sprecher

**Running title:** Probing ethanol and magnesium chloride as anaesthetics for *Loligo vulgaris*

**Keywords:** anaesthesia; cephalopods; embryos; *Loligo vulgaris*

**Supplementary Table 1**. Summary of the p values after Repeated measures ANOVA on heart rate detected in *L. vulgaris* embryos during different phases of the experiment (t0, t1, t2) and exposed to different concentrations of EtOH, the MgCl_2_ and control group.

|  | **T0 and T1** | **T0 and T2** | **T1 and T2** |
| --- | --- | --- | --- |
| **EtOH group** | **Significant** | **Not Significant** | **Significant** |
| 3% EtOH | <0.001 | 1 | <0.001 |
| 2.5% EtOH | 0.003 | 0.457 | 0.792 |
| 2% EtOH | 0.113 | 0.977 | 0.939 |
| Control | 1 | 1 | 1 |

|  | **T0 and T1** | **T0 and T2** | **T1 and T2** |
| --- | --- | --- | --- |
| **MgCl_2_ group** | **Significant** | **Not Significant** | **Significant** |
| 1.8% MgCl_2_ | <0.001 | <0.001 | 0.995 |
| 1.5% MgCl_2_ | <0.001 | 0.999 | <0.001 |
| 1% MgCl_2_ | 0.002 | 0.210 | 0.115 |
| Control | 1 | 1 | 1 |

**Supplementary Table 2**. Summary of the p values after Repeated measures ANOVA on respiratory rate detected in *L. vulgaris* embryos during different phases of the experiment (t0, t1, t2) and exposed to different concentrations of EtOH, the MgCl_2_ and control group.

|  | **T0 and T1** | **T0 and T2** | **T1 and T2** |
| --- | --- | --- | --- |
| **EtOH** | **Significant** | **Not Significant** | **Significant** |
| 3% EtOH | <0.001 | 0.431 | <0.001 |
| 2.5% EtOH | <0.001 | 0.500 | 0.087 |
| 2% EtOH | 0.003 | 0.999 | 0.192 |
| Control | 1.000 | 1 | 1 |

|  | **T0 and T1** | **T0 and T2** | **T1 and T2** |
| --- | --- | --- | --- |
| **MgCl_2_** | **Significant** | **Not Significant** | **Significant** |
| 1.8% MgCl_2_ | <0.001 | <0.001 | 1 |
| 1.5% MgCl_2_ | <0.001 | 0.956 | <0.001 |
| 1% MgCl_2_ | 0.079 | 0.939 | 0.178 |
| Control | 1 | 1.000 | 1 |

**Supplementary Table 3**. Paired t-test statistics considering time until loss of buoyancy (TL B) and time to recovery of buoyancy (TR B) of *L. vulgaris* embryos during different phases of the experiment (t0, t1, t2) and exposed to different concentrations of EtOH, the MgCl_2_ and control group.


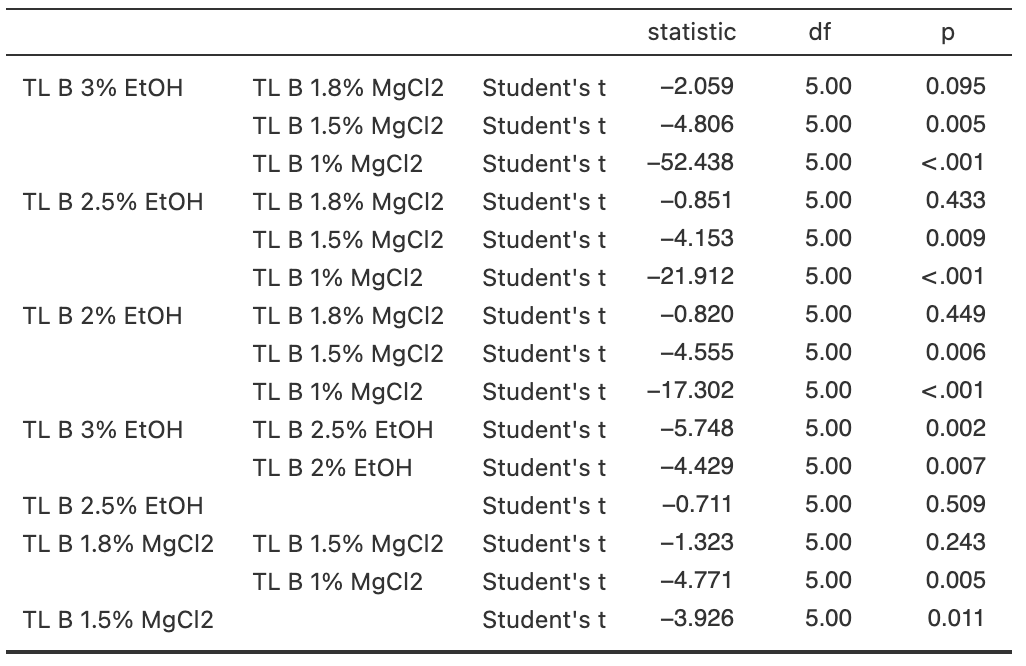


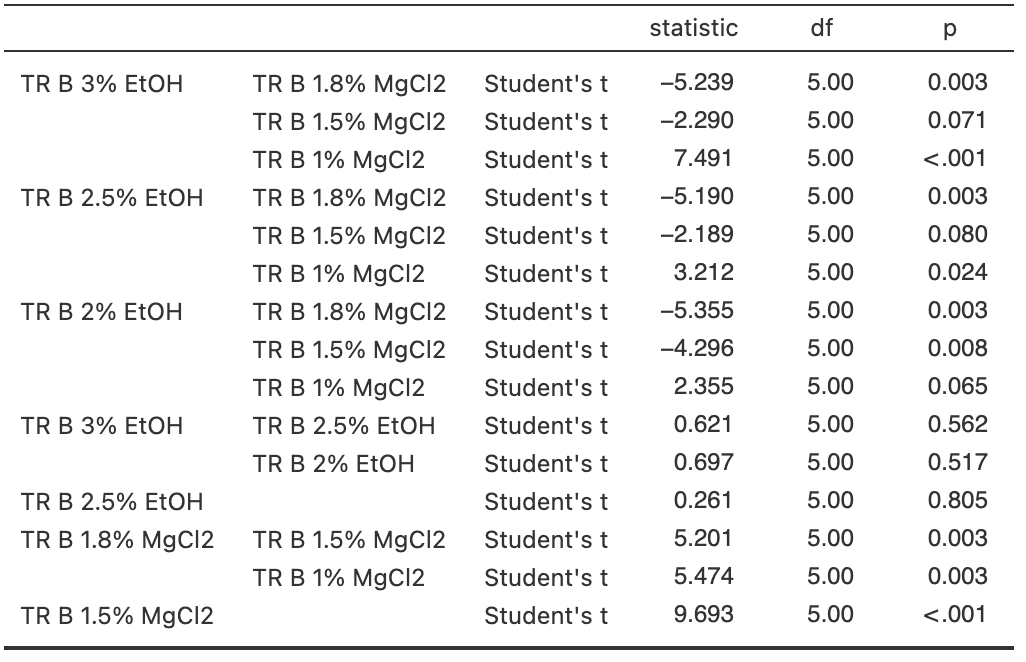


**Supplementary Table 4**. Paired t-test statistics considering time until loss of chromatophore activity (TL CR) and time to recovery of chromatophore tone (TR CR) of *L. vulgaris* embryos during different phases of the experiment (t0, t1, t2) and exposed to different concentrations of EtOH, the MgCl_2_ and control group.


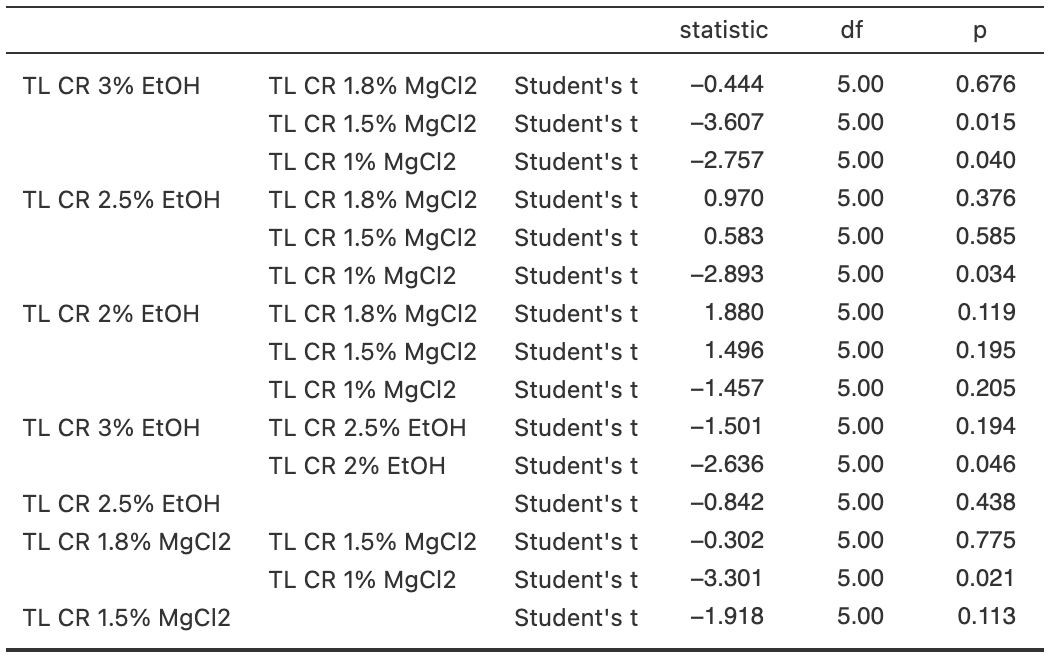


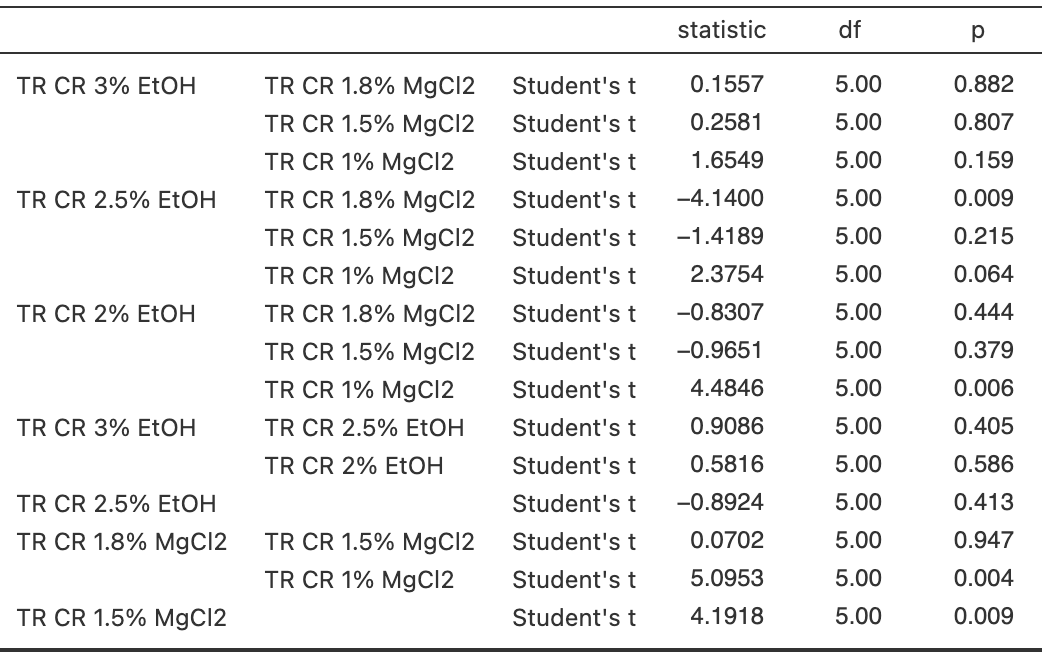


**Supplementary Table 5**. Paired t-test statistics considering time until loss of Tentacles/arms response “TL T/A R” and time to recovery of Tentacles/arms response “TR T/A R” of *L. vulgaris* embryos during different phases of the experiment (t0, t1, t2) and exposed to different concentrations of EtOH, the MgCl_2_ and control group.


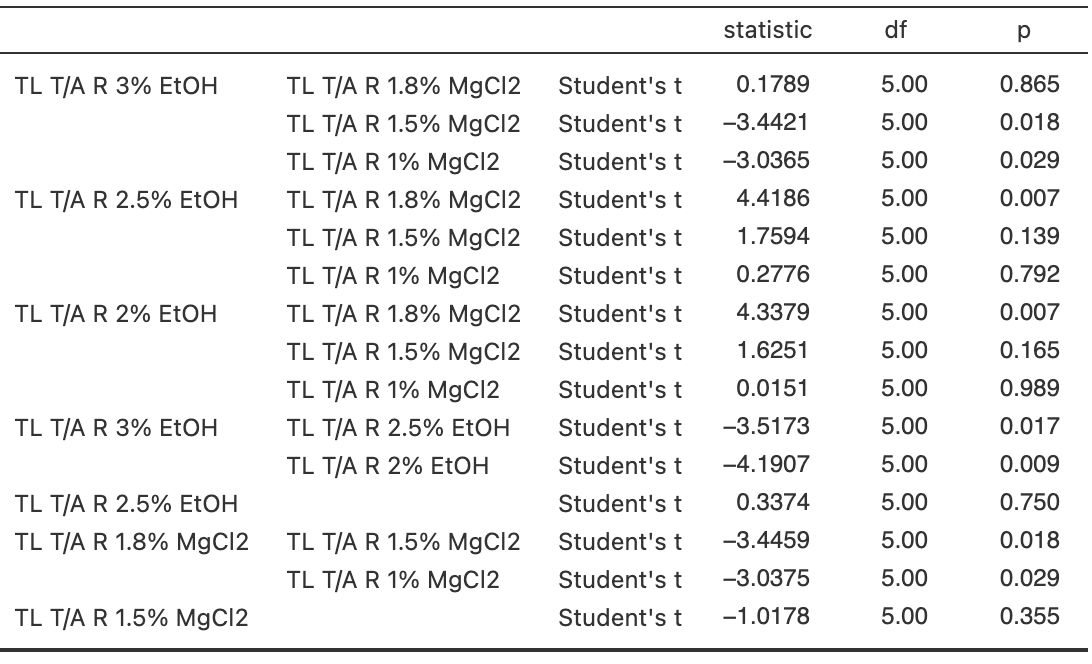


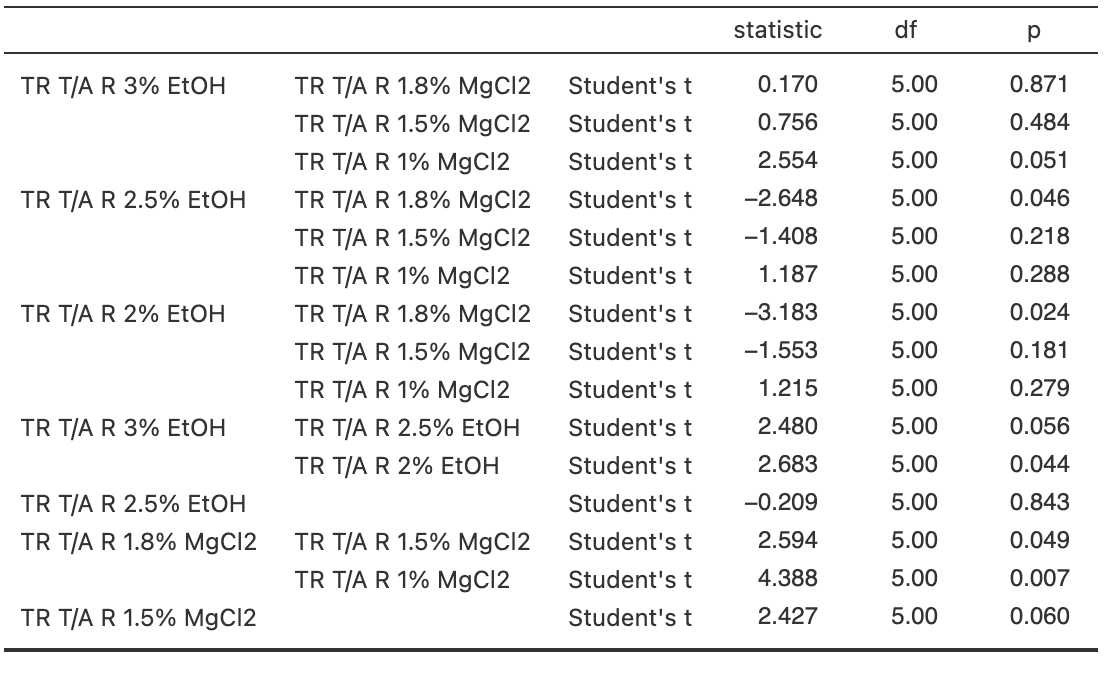

Supplement: Supplementary file 1 [file DataSheet1.docx]
